# WWP1 Deficiency Alleviates Cardiac Remodeling Induced by Simulated Microgravity

**DOI:** 10.3389/fcell.2021.739944

**Published:** 2021-10-18

**Authors:** Guohui Zhong, Dingsheng Zhao, Jianwei Li, Zifan Liu, Junjie Pan, Xinxin Yuan, Wenjuan Xing, Yinglong Zhao, Shukuan Ling, Yingxian Li

**Affiliations:** ^1^The Key Laboratory of Aerospace Medicine, Ministry of Education, Fourth Military Medical University, Xi’an, China; ^2^State Key Laboratory of Space Medicine Fundamentals and Application, China Astronaut Research and Training Center, Beijing, China; ^3^Department of Cardiovascular Medicine, Chinese PLA General Hospital & Chinese PLA Medical School, Beijing, China; ^4^Medical College of Soochow University, Soochow University, Suzhou, China; ^5^Key Laboratory of Molecular and Cellular Biology of Ministry of Education, College of Life Sciences, Hebei Normal University, Shijiazhuang, China

**Keywords:** WWP1, simulated microgravity, cardiac remodeling, DVL2, HDAC4

## Abstract

Cardiac muscle is extremely sensitive to changes in loading conditions; the microgravity during space flight can cause cardiac remodeling and function decline. At present, the mechanism of microgravity-induced cardiac remodeling remains to be revealed. WW domain-containing E3 ubiquitin protein ligase 1 (WWP1) is an important activator of pressure overload-induced cardiac remodeling by stabilizing disheveled segment polarity proteins 2 (DVL2) and activating the calcium-calmodulin-dependent protein kinase II (CaMKII)/histone deacetylase 4 (HDAC4)/myocyte-specific enhancer factor 2C (MEF2C) axis. However, the role of WWP1 in cardiac remodeling induced by microgravity is unknown. The purpose of this study was to determine whether WWP1 was also involved in the regulation of cardiac remodeling caused by microgravity. Firstly, we detected the expression of WWP1 and DVL2 in the heart from mice and monkeys after simulated microgravity using western blotting and immunohistochemistry. Secondly, WWP1 knockout (KO) and wild-type (WT) mice were subjected to tail suspension (TS) to simulate microgravity effect. We assessed the cardiac remodeling in morphology and function through a histological analysis and echocardiography. Finally, we detected the phosphorylation levels of CaMKII and HDAC4 in the hearts from WT and WWP1 KO mice after TS. The results revealed the increased expression of WWP1 and DVL2 in the hearts both from mice and monkeys after simulated microgravity. WWP1 deficiency alleviated simulated microgravity-induced cardiac atrophy and function decline. The histological analysis demonstrated WWP1 KO inhibited the decreases in the size of individual cardiomyocytes of mice after tail suspension. WWP1 KO can inhibit the activation of the DVL2/CaMKII/HDAC4 pathway in the hearts of mice induced by simulated microgravity. These results demonstrated WWP1 as a potential therapeutic target for cardiac remodeling and function decline induced by simulated microgravity.

## Introduction

Our organ systems have evolved to work under 1*g* environment. It is not yet clear what effects will be produced by long-term exposure to low-gravity environments and how these effects will be manifested at the cellular and molecular levels (Walls et al., 2020). Since the first human went into space, cardiac health has been a major concern of the world’s space agencies; however, the clinical evidence for decreased function and unmasking of asymptomatic cardiovascular disease is limited.

The heart is essentially a muscle, whose basic function is to provide the circulatory system with pump power (Maillet et al., 2013). The cardiac muscle is very sensitive to changes in loading conditions, usually caused by pathological or physiological stimulation (Hill and Olson, 2008). A variety of stimuli can induce the heart to grow or shrink. Exercise, pregnancy, and postnatal growth promote physiologic growth of the heart (Zhong et al., 2016). Long-term pressure overload leads to pathological hypertrophy and heart failure (Hill and Olson, 2008). Cardiac atrophy was a complication for prolonged microgravity during space flight, long-term bed rest, and mechanical unloading with a ventricular assist device (Levine et al., 1997; Hill and Olson, 2008; Westby et al., 2016; Ling et al., 2018). The tail suspension of mice or rats is widely utilized to study the effects of microgravity (Respress et al., 2014), and head-down tilt bed rest model for non-human primate rhesus monkeys or human volunteers is also a classical ground-based model of microgravity (Wang et al., 2012; Chen et al., 2016; Ling et al., 2018). Our early researches also demonstrated that simulated microgravity induced cardiac atrophy and function decline of mice and rhesus monkeys (Zhong et al., 2016; Ling et al., 2018).

Cardiac muscle is extremely sensitive to changes in loading conditions (Tuday and Berkowitz, 2007; Ling et al., 2018). When exposed to microgravity during space flight, there are various changes in cardiac structure and function (Hill and Olson, 2008; Hughson et al., 2018). Microgravity can cause a chronic decrease in metabolic demand and oxygen uptake, thereby reducing the demand for cardiac output, leading to cardiac atrophy and decline in cardiac function (Dorfman et al., 2007; Zhong et al., 2016). There are many important factors that can regulate cardiac remodeling caused by various stimuli, including disheveled segment polarity proteins 2 (DVL2), calcium-calmodulin-dependent protein kinase II (CaMKII), and histone deacetylase 4 (HDAC4; Ling et al., 2012; Zhao et al., 2021). Our previous studies have shown that pathological cardiac remodeling signals (such as HDAC4) were activated in the heart of mice after tail suspension, which may lead to cardiac remodeling and decline in function (Zhong et al., 2016; Ling et al., 2018). Walls et al. (2020) found that exposure to microgravity aboard the International Space Station (ISS) caused heart dysfunction in a fly cardiac model: hearts are less contractile and exhibit changes in genes and proteins that maintain heart structure and function; among which, it is worth noting that proteasome gene expression was upregulated, suggesting that the regulation of protein homeostasis mediated by ubiquitination modification plays an important role in myocardial remodeling caused by microgravity.

WW domain-containing E3 ubiquitin protein ligase 1 (WWP1) is a C2-WW-HECT-type ubiquitin E3 ligase containing an N-terminal C2 domain, four tandem WW domains, and a C-terminal catalytic HECT domain for ubiquitin transferring (Zhi and Chen, 2012). WWP1 regulates a variety of cellular biological processes including protein trafficking and degradation, transcription, and signaling by functioning as the E3 ligase (Peschiaroli et al., 2010; Zhao et al., 2012, 2021; Shu et al., 2013; Lee et al., 2019). Cardiomyocyte-specific overexpression of WWP1 is detrimental to the heart for it can induce arrhythmia and hypertrophy (Basheer et al., 2015). Also, in our previous research, we identified WWP1 as a novel regulator of pathological cardiac hypertrophy and heart failure (Zhao et al., 2021). Pressure overload-induced heart hypertrophy was relieved in WWP1 knockout (KO) mice (Zhao et al., 2021). Mechanically, WWP1 stabilized DVL2 by promoting its K27-linked polyubiquitination and regulated cardiac remodeling through DVL2/CaMKII/HDAC4/myocyte-specific enhancer factor 2C (MEF2C) pathway (Zhao et al., 2021). However, the role of WWP1 in cardiac remodeling induced by microgravity was unknown.

In this study, we found WWP1 protein levels were increased in the heart of mice and rhesus monkeys after simulated microgravity. WWP1 deficiency alleviated simulated microgravity-induced cardiac atrophy, function decline, and pathological signal activation in mice, and WWP1 has potential as a therapeutic target for cardiac remodeling and function decline induced by space flight.

## Materials and Methods

### Animal Experiments

The experimental procedures in mice and the protocol used in this study were approved by the Animal Care and Use Committee of China Astronaut Research and Training Center. All animal studies were performed according to approved guidelines for the use and care of live animals (Guideline on Administration of Laboratory Animals released in 1988 and 2006, Guideline on Humane Treatment of Laboratory Animals from China, and also referring to European Union guideline 2010/63).

The healthy rhesus monkeys with body weight of 5–8 kg and 4–8 years old were purchased from Beijing Xieerxin Biology Resource (Beijing, China). The monkeys were maintained at a 10-degree head-down tilt position for 6 weeks. The whole process was supervised and monitored 24 h/day.

WW domain-containing E3 ubiquitin protein ligase 1 knockout mice were bought from the Model Animal Research Center of Nanjing University, as reported previously. All wild-type (WT) and WWP1 KO mice used in this study were bred and housed at the specific pathogen-free (SPF) Animal Research Center of China Astronaut Research and Training Center (12:12-h light–dark cycle, temperature: 23°C). All the experiments were repeated three times and were performed with homozygous WWP1-deficient mice (3 months old) and age-matched WT littermates. The simulated microgravity procedure was by elevated tail suspension, as described before (Zhong et al., 2016). Briefly, the 3-month-old mice were maintained in individual cage and suspended with a strip placed around the proximal two thirds of the tail and linked to a chain hanging from a pulley. The mice were elevated to an angle of 30° from the floor with only the forelimbs touching the floor, which allowed the mice to move and access food and water freely. The height of tail suspension was modulated according to the length and weight of each mouse to prevent the hindlimb from touching the ground, which allowed free range of movement around the cage while achieving the desired effects. The tail suspension was maintained for 6 weeks. Control mice of the same strain background and the age-matched littermates were instrumented and monitored in the identical cage conditions without tail suspension.

### Echocardiography

As described in our previous studies (Ling et al., 2012), animals were lightly anesthetized with 2,2,2-tribromoethanol (0.2 ml/10 g body weight of a 1.2% solution) and set in a supine position. Two-dimensional (2D) guided M-mode echocardiography was performed using a high-resolution imaging system (Vevo 1100, Visual-Sonics Inc., Toronto, ON, Canada). Two-dimensional images are recorded in parasternal long- and short-axis projections with guided M-mode recordings at the midventricular level in both views. Left ventricular (LV) cavity size and wall thickness are measured in at least three beats from each projection. Averaged LV wall thickness [anterior wall (AW) and posterior wall (PW) thickness] and internal dimensions at diastole and systole (LVIDd and LVIDs, respectively) are measured. LV fractional shortening [(LVIDd - LVIDs)/LVIDd] and LV mass {LV mass = 1.053 × [(LVID;d + LVPW;d + LVAW;d)^3^ - LVID;d^3^]} are calculated from the M-mode measurements. LV ejection fraction (EF) was calculated from the LV cross-sectional area (2-D short-axis view) using the equation LV%EF = (LV Vol;d - LV Vol;s)/LV Vol;d × 100%. The studies and analysis were performed blinded as to experimental groups.

### RNA Extraction and Real-Time PCR

Total RNA was extracted from heart tissues with TRIzol reagent according to the manufacturer’s protocol. The RNA (1 μg/sample) was reverse transcribed into cDNA, and real-time quantitative polymerase chain reaction (Q-PCR) was performed using a SYBR Green PCR Kit (Takara) in a Light Cycler (Eppendorf). The expression level of each gene was normalized to that of Gapdh, which served as an endogenous internal control. Primers (synthesized by BGI, China) for *BNP*, *Col1*α*1*, Col3α1, and *Gapdh* were as follows:

*BNP* forward primer: 5′-TGTTTCTGCTTTTCCTTT ATCTG-3′,
*BNP* reverse primer: 5′-TCTTTTTGGGTGTTCTTT TGTGA-3′;
*Col1*α*1* forward primer: 5′-CTGACTGGAAGAGCGGA GAGT-3′,
*Col1*α*1* reverse primer: 5′-AGACGGCTGAGTAGGGA ACAC-3′;
*Col3*α*1* forward primer: 5′-ACGTAAGCACTGGTGG ACAG-3′,
*Col3*α*1* reverse primer: 5′-CAGGAGGGCCATAGC TGAAC-3′; and
*Gapdh* forward primer: 5′-ACTCCACTCACGGCAAA TTCA-3′,
*Gapdh* reverse primer: 5′-GGCCTCACCCCATTT GATG-3′.

### Protein Extraction and Western Blot

Heart tissues from mice or rhesus monkeys were crushed by a homogenizer (Power Gen125, Fisher Scientific) and then lysed in lysis buffer (50 mM Tris, pH 7.5, 250 mM NaCl, 0.1% sodium dodecyl sulfate, 2 mM dithiothreitol, 0.5% NP-40, 1 mM PMSF, and protease inhibitor cocktail) on ice for 30 min. Protein fractions were collected by centrifugation at 13,000*g* at 4°C for 15 min. Protein samples were separated by 10% SDS–PAGE and transferred to nitrocellulose membranes. The membranes were blocked with 5% bovine serum albumin and incubated with specific antibodies overnight. Antibodies used were as follows: WWP1 (1:1,000, Abcam, United States, #ab43791), DVL2 (1:1,000, Cell Signaling Technology, United States, #3224S), CaMKII (1:1,000, GeneTex, United States, GTX111401), p-CaMKII (1:1,000, T287, GeneTex, United States, GTX52342), HDAC4 (1:1,000, Cell Signaling Technology, United States, #5392), p-HDAC4 (1:1,000, S246, Cell Signaling Technology, United States, #3443), and GAPDH (1:2,000, Abways Technology, China, #AB0036).

### Histological Analysis

Sections for hematoxylin and eosin (H&E) and Masson trichrome staining were generated from paraffin-embedded hearts. Frozen sections were used to visualize cardiomyocyte cell membranes by staining with FITC-conjugated wheat germ agglutinin (Sigma-Aldrich, United States), as described before. For immunohistochemical staining, sections were deparaffinized in xylene and rehydrated. Antigen retrieval was performed with protease K at 37°C for 15 min. A solution of 3% H_2_O_2_ was used to block the activity of endogenous peroxidase. The sections were then incubated overnight at 4°C with WWP1 (1/100, Abcam, #ab43791) or DVL2 antibody (1/100, Cell Signaling Technology, #3224S). After three washes in PBS, biotinylated secondary antibodies were then added and incubated for 1 h at room temperature, followed by color development with DAB kit (ZSGB-Bio). Negative control experiments were done by omitting the primary antibodies. The sections were examined using a microscope (ECLIPSE Ci-S, Nikon).

### Statistical Analysis

Data are presented as mean ± SEM. Statistical analysis for comparison of two groups was performed using two-tailed unpaired Student’s *t*-test. Statistical differences among groups were analyzed by one-way analysis of variance (ANOVA) or two-way ANOVA (if there were two factor levels), followed by Bonferroni’s *post hoc* test to determine group differences in the study parameters. Pearson correlation coefficients (*r*^2^) was performed to assess the correlation between two variables. All statistical analyses were performed with Prism software (GraphPad Prism for Windows, version 9.0, Nashville, TN, United States). Differences were considered significant at ^∗^*P* < 0.05, ^∗∗^*P* < 0.01, and ^∗∗∗^*P* < 0.001.

## Results

### Characterization of WW Domain-Containing E3 Ubiquitin Protein Ligase 1 Expression During Simulated Microgravity-Induced Cardiac Remodeling

**FIGURE 1:**
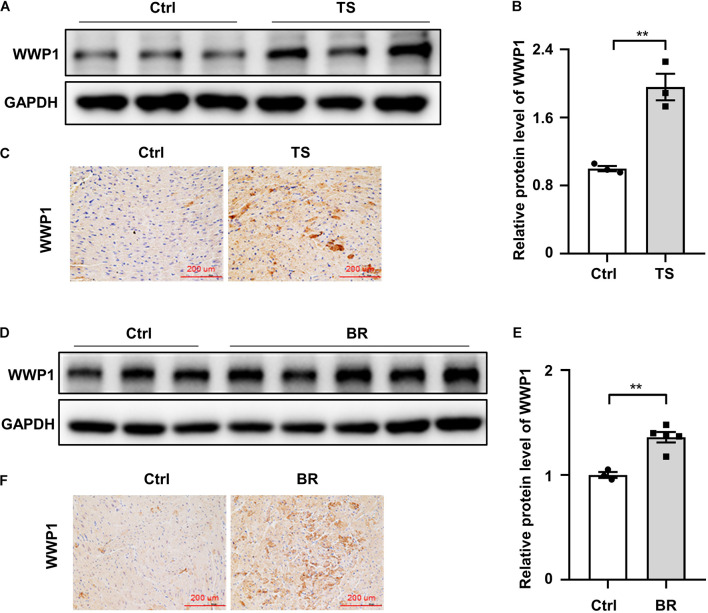
WW domain-containing E3 ubiquitin protein ligase 1 expression changes in the hearts of mice and rhesus monkeys after simulated microgravity. **(A)** Representative western blotting analysis of WWP1 expression in cardiac extracts of adult mice following tail suspension (TS) for 6 weeks (*n* = 3 for each group). **(B)** Quantification of WWP1 protein levels of **(A)**. **(C)** Immunohistochemistry for WWP1 (brown) in paraffin section from mouse hearts at 6 weeks after tail suspension. Scale bars, 200 μm. **(D)** Representative western blotting analysis of WWP1 expression in cardiac extracts of adult rhesus monkeys following bed rest (BR) for 6 weeks (Ctrl, *n* = 3; BR, *n* = 5). **(E)** Quantification of WWP1 protein levels of **(D)**. **(F)** Immunohistochemistry for WWP1 (brown) in paraffin section from rhesus monkey hearts at 6 weeks after bed rest. Scale bars, 200 μm. Data represent the means ± SEM. *P < 0.05, **P < 0.01. WWP1, WW domain-containing E3 ubiquitin protein ligase 1; TS, tail suspension; BR, bed rest.

To assess the potential role of WWP1 in cardiac remodeling induced by simulated microgravity, hearts from mice after 6 weeks of tail suspension were assessed for WWP1 expression. As shown in Figures 1A–C, western blotting and immunohistochemistry revealed that the protein level of WWP1 was significantly increased in the hearts of mice after tail suspension. Moreover, we detected WWP1 expression in the hearts of rhesus monkeys after 6 weeks of bed rest and found that the protein levels of WWP1 increased in the heart of rhesus monkeys after bed rest (Figures 1D–F). These results suggested that simulated microgravity increase the protein level of WWP1, which was an activator of pathological cardiac remodeling.

### WW Domain-Containing E3 Ubiquitin Protein Ligase 1 Deficiency Protects Against Simulated Microgravity-Induced Cardiac Function Decline

**FIGURE 2:**
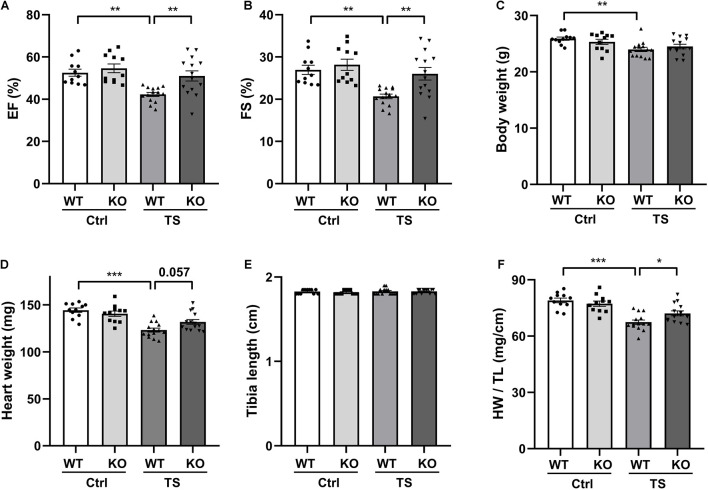
WW domain-containing E3 ubiquitin protein ligase 1 knock out alleviates simulated microgravity-induced cardiac function decline. **(A,B)** Echocardiographic assessment of ejection fraction (EF) and fractional shortening (FS) of WT and WWP1 knockout (KO) mice after 6 weeks of tail suspension. **(C–F)** Body weight, heart weight, tibia length, and the ratio of heart weight to tibia length of wild-type (WT) and WWP1 KO mice after 6 weeks of tail suspension. Data represent the means ± SEM. *P < 0.05, **P < 0.01, ***P < 0.001. Ctrl, control; TS, tail suspension; WT, wild-type mice; KO, knockout.

To further investigate the potential role of WWP1 in cardiac remodeling induced by simulated microgravity, we compared the responses of WT and WWP1 KO mice to TS. WT and KO littermates at 3 months of age were subjected to tail suspension for 6 weeks. The relevant control group were treated equally, except for the tail suspension. Cardiac function was calculated by echocardiography (Figures 2A,B), and body weight, heart weight (HW), and tibia length (TL) were recorded (Figures 2C–E). All echocardiographic measurements were made while the heart rates of mice were maintained at 450–550 beats/min. The two-way ANOVA reports showed that there was a statistically significant interaction between the effects of treatment and genotype on EF (treatment, *p* = 0.0005; genotype, *p* = 0.0027; and interaction, *p* = 0.0351), FS (treatment, *p* = 0.0007; genotype, *p* = 0.0247; and interaction, *p* = 0.0364), and the ratio of heart weight to tibia length (HW/TL) (treatment, *p* < 0.0001; genotype, *p* = 0.2631; and interaction, *p* = 0.0168). Compared with the control group, left ventricular EF and fractional shortening (FS) decreased significantly in WT mice after 6 weeks of tail suspension, whereas no such changes in KO mice are apparent (Figures 2A,B). Body weight and heart weight decreased significantly in WT mice (Figures 2C,D), and tibia length did not change in either WT or KO mice after TS (Figure 2E). Moreover, HW/TL decreased significantly in WT but not in WWP1 KO mice after TS (Figure 2F), suggesting that WWP1 knockout protects against simulated microgravity-induced cardiac atrophy and function decline.

### Left Ventricular Structure of Wild-Type and Knockout Mice Following Tail Suspension

**FIGURE 3:**
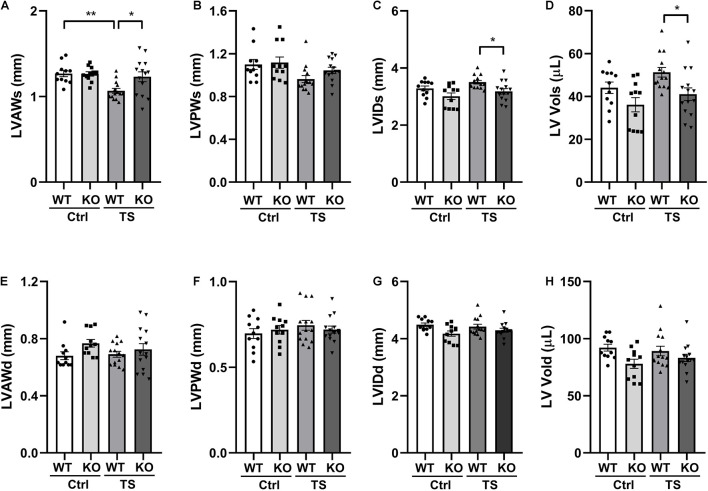
The left ventricular structure of WT and WWP1 KO mice after tail suspension. **(A–H)** Quantitative analysis of the diastolic and systolic left ventricular posterior wall thickness (LVPWd and LVPWs), LV anterior wall thickness (LVAWd and LVAWs), LV internal diameter (LVIDd and LVIDs), and LV volume (LV Vold and LV Vols) from WT and WWP1 KO mice by echocardiography following tail suspension. Data represent the means ± SEM. *P < 0.05, **P < 0.01. Ctrl, control; TS, tail suspension; WT, wild-type mice; KO, knockout.

To validate the influence of WWP1 KO in the heart structure after tail suspension, we performed transthoracic echocardiography to determine the left ventricular structure of WT and KO mice that were subjected to tail suspension (Figure 3). There was a statistically significant interaction between the effects of treatment and genotype on the end-diastolic left ventricular anterior wall thickness (LVAWs) (treatment, *p* = 0.0064; genotype, *p* = 0.0577; and interaction, *p* = 0.046). Compared with control, the LVAWs of WT mice was decreased following tail suspension; however, the LVAWs of WWP1 KO mice after tail suspension did not change, and the value was higher than that in WT mice after tail suspension (Figure 3A). The end-systolic left ventricular posterior wall thickness (LVPWs) showed a decreasing trend in WT mice but not in KO mice after tail suspension (Figure 3B). In the TS group, both the LVIDd (Figure 3C) and the end-systolic LV volume (LV Vols) (Figure 3D) of KO mice are lower than those of WT mice. Meanwhile, the end-diastolic anterior wall thickness (LVAWd) (Figure 3E), the end-diastolic left ventricular posterior wall thickness (LVPWd) (Figure 3F), the LVIDs (Figure 3G), and the end-diastolic LV volume (LV Vold) (Figure 3H) did not change in the WT and KO mice after tail suspension. These data indicated that WWP1 KO alleviated the decreasing LVAWs induced by tail suspension.

### WW Domain-Containing E3 Ubiquitin Protein Ligase 1 Knockout Alleviates Simulated Microgravity Induced-Cardiac Remodeling

**FIGURE 4:**
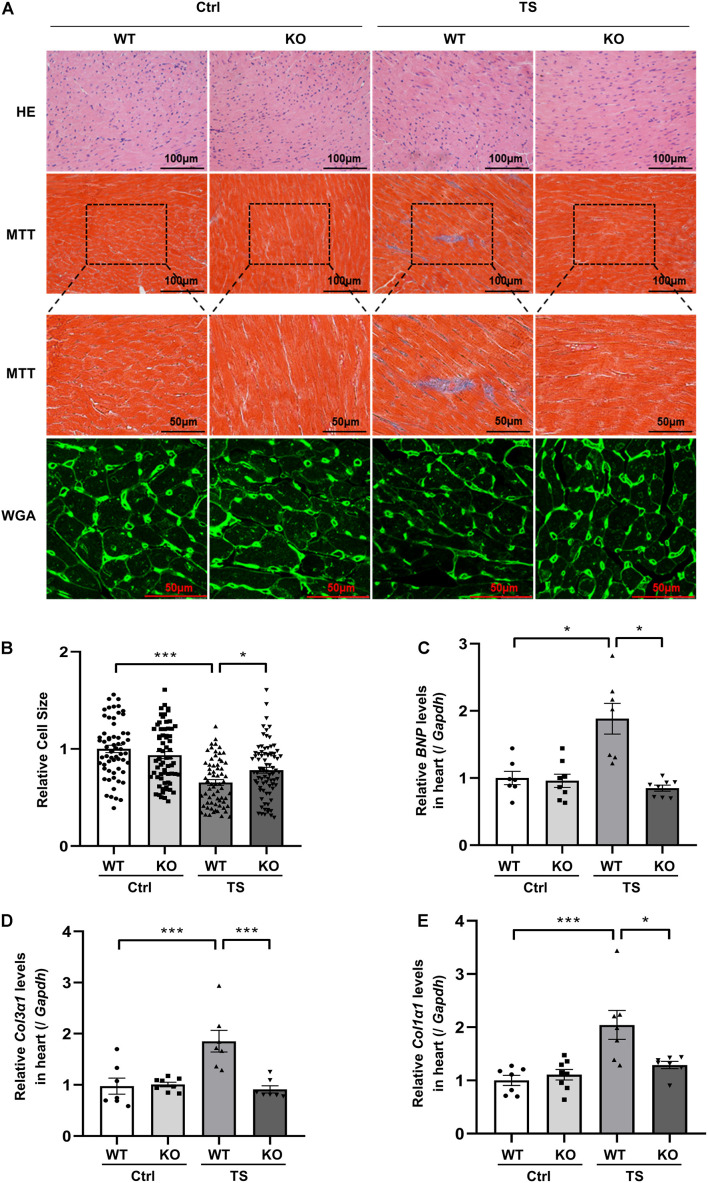
WW domain-containing E3 ubiquitin protein ligase 1 knockout protects against simulated microgravity induced-cardiac atrophy. **(A)** Hematoxylin and eosin (H&E)-stained sections of the hearts from WT and WWP1 KO mice after 6 weeks of tail suspension. Sections of the hearts are stained with Masson trichrome (MTT) to detect fibrosis (blue). Wheat germ agglutinin (WGA) staining is used to demarcate cell boundaries. **(B)** The cardiomyocyte cross-sectional area was measured from 7-μm-thick heart sections that have been stained with WGA by ImageJ software (NIH). Only myocytes that were round were included in the analysis. **(C–E)** The mRNA levels of *BNP*, *Col1*α*1*, and *Col3*α*1* were analyzed by Q-PCR from WT and WWP1 KO mice after 6 weeks of tail suspension. The relative abundance of transcripts were quantified and normalized to *Gapdh*. Data represent the means ± SEM, *P < 0.05, **P < 0.01, ***P < 0.001. *Col1*α*1*, alpha-1 type I collagen; *Col3*α*1*, alpha-1 type III collagen; BNP, brain natriuretic peptide; Q-PCR, real-time quantitative polymerase chain reaction.

To address the effect of WWP1 knockout on cardiac remodeling induced by tail suspension, hearts from WT and KO mice were assessed for changes in morphology and cardiac remodeling gene expression. As shown in Figure 4A, Masson trichrome staining (MTT) showed a deeper staining of collagen in the heart of WT mice after tail suspension, but WWP1 KO mice after tail suspension had no obvious change.

Wheat germ agglutinin (WGA) staining was used to demarcate cell boundaries of cardiomyocytes; compared with control, relative cell size was decreased in WT mice after tail suspension; however, WWP1 KO can withstand the effect of tail suspension (Figures 4A,B). The results of Q-PCR showed that transcripts for the pathological cardiac remodeling genes BNP, Col1α1, and Col3α1 were significantly increased in the hearts of WT mice after tail suspension, and WWP1 KO significantly attenuated the increase of cardiac remodeling gene expression induced by simulated microgravity (Figures 4C–E). What is more, there was a statistically significant interaction between the effects of treatment and genotype on cell size (treatment, *p* < 0.0001; genotype, *p* = 0.3468; and interaction, *p* = 0.0048), BNP (treatment, *p* = 0.0063; genotype, *p* = 0.0003; and interaction, *p* = 0.0008), Col1α1 (treatment, *p* = 0.0005; genotype, *p* = 0.0446; and interaction, *p* = 0.0093), and Col3α1 (treatment, *p* = 0.0071; genotype, *p* = 0.0023; and interaction, *p* = 0.0012). These results suggested that WWP1 KO alleviates simulated microgravity-induced cardiac remodeling.

### Disheveled Segment Polarity Proteins 2 Positively Correlates With WW Domain-Containing E3 Ubiquitin Protein Ligase 1 in the Heart of Mice and Rhesus Monkeys After Simulated Microgravity

**FIGURE 5:**
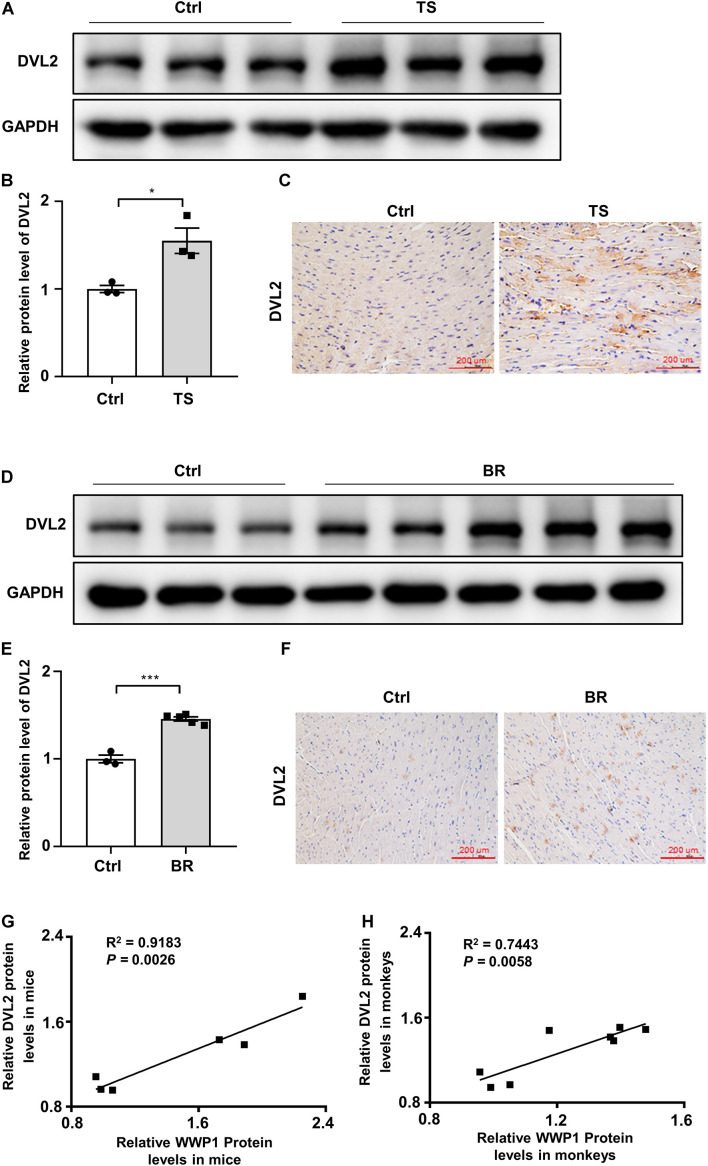
Disheveled segment polarity proteins 2 expression changes in the hearts of mice and rhesus monkeys after simulated microgravity. **(A)** Representative western blotting analysis of DVL2 expression in cardiac extracts of adult mice following tail suspension (TS) for 6 weeks (*n* = 3 for each group). **(B)** Quantification of DVL2 protein levels of **(A)**. **(C)** Immunohistochemistry for DVL2 (brown) in paraffin section from mouse hearts at 6 weeks after tail suspension. Scale bars, 200 μm. **(D)** Representative western blotting analysis of DVL2 expression in cardiac extracts of adult rhesus monkeys following bed rest (BR) for 6 weeks (Ctrl, *n* = 3; BR, *n* = 5). **(E)** Quantification of DVL2 protein levels of **(D)**. **(F)** Immunohistochemistry for DVL2 (brown) in a paraffin section from rhesus monkey hearts at 6 weeks after bed rest. **(G)** Pearson correlation coefficients between DVL2 and WWP1 protein levels in the heart of mice following tail suspension. **(H)** Pearson correlation coefficients between DVL2 and WWP1 protein levels in the heart of rhesus monkeys following bed rest. Scale bars, 200 μm. Data represent the means ± SEM. *P < 0.05, ***P < 0.001. DVL2, disheveled segment polarity protein 2; TS, tail suspension; BR, bed rest.

Our previous research found that WWP1 can participate in the regulation of myocardial remodeling by stabilizing DVL2 (Zhao et al., 2021). In order to verify whether the above mechanism also exists in the myocardial remodeling caused by simulated microgravity, we analyzed the protein level of DVL2 in the heart of mice subjected to tail suspension and rhesus monkeys subjected to bed rest. The protein level of DVL2 significantly increased (Figures 5A–F) and was significantly positively correlated with the expression of WWP1 in the heart of mice and rhesus monkeys subjected to simulated microgravity (Figures 5G,H), suggesting that WWP1 is correlated with DVL2 protein stability during simulated microgravity-induced cardiac remodeling.

### WW Domain-Containing E3 Ubiquitin Protein Ligase 1 Regulates Simulated Microgravity-Induced Cardiac Remodeling *via* Disheveled Segment Polarity Proteins 2/Calcium-Calmodulin-Dependent Protein Kinase II/Histone Deacetylase 4 Axis

**FIGURE 6:**
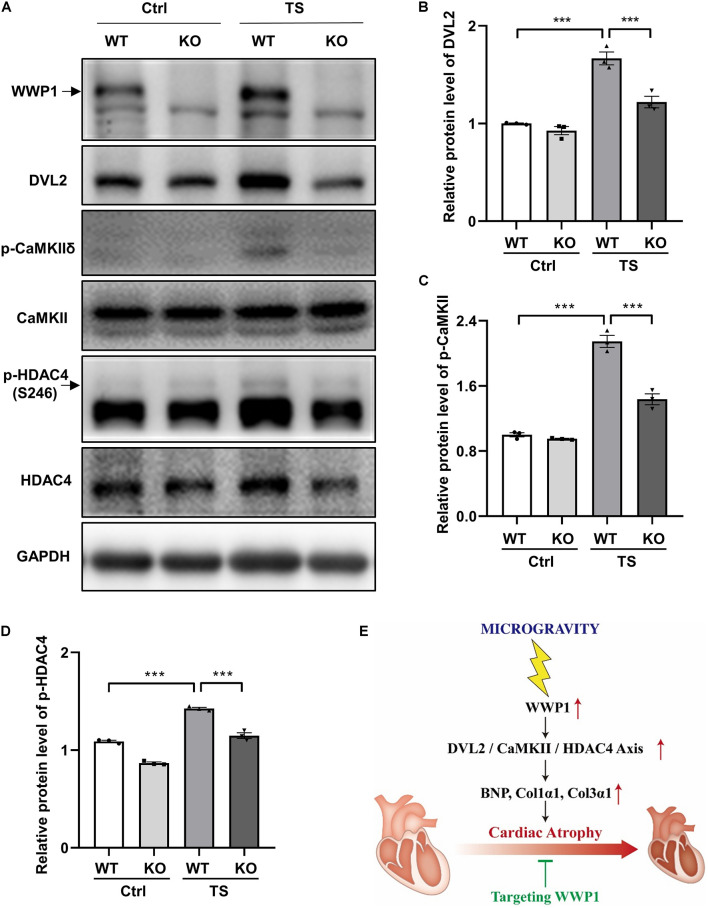
WW domain-containing E3 ubiquitin protein ligase 1 knockout inhibits DVL2/CaMKII/HDAC4 signal in mice heart after simulated microgravity. **(A)** Representative western blots for WWP1, DVL2, and CaMKIIδ and phosphorylation at Thr287 and HDAC4 and phosphorylation at Ser246 in the hearts from WT and WWP1 KO mice after 6 weeks of tail suspension. GAPDH levels served as a loading control. **(B–D)** Quantification analysis of corresponding protein in **(A)**. **(E)** Model of WWP1 function in simulated microgravity-induced cardiac remodeling signaling. Data represent the means ± SEM. ***P < 0.001. WWP1, WW-domain containing E3 ubiquitin protein ligase 1; DVL2, disheveled segment polarity protein 2; CaMKII, calcium/calmodulin-dependent protein kinase II; HDAC4, histone deacetylase 4; TS, tail suspension.

To gain more insights into the effect of WWP1 KO on the signaling pathways involved in the cardiac remodeling induced by simulated microgravity, we examined the protein level of DVL2, phosphorylation levels of CaMKII, and phosphorylation levels of HDAC4 in the heart tissues of WT and KO mice after tail suspension (Figure 6A). The two-way ANOVA reports showed that there were statistically significant interactions between the effects of treatment and genotype on the protein levels of DVL2 (treatment, *p* < 0.0001; genotype, *p* = 0.0007; and interaction, *p* = 0.0051) and the phosphorylation levels of CaMKII at Thr287 (treatment, *p* < 0.0001; genotype, *p* < 0.0001; and interaction, *p* = 0.0002). As shown in Figures 6B–D, a quantification analysis revealed that the levels of DVL2, p-CaMKII, and p-HDAC4 were significantly increased in WT mice after tail suspension, and WWP1 KO significantly attenuated the increase of cardiac remodeling-related signals induced by simulated microgravity. These results indicated that WWP1 regulates simulated microgravity-induced cardiac remodeling *via* the DVL2/CaMKII/HDAC4 axis (Figure 6E).

## Discussion

Here, we identified WWP1 as a novel regulator of simulated microgravity-induced cardiac remodeling. WWP1 involved in the regulation of simulated microgravity-induced cardiac remodeling through the DVL2/CaMKII/HDAC4/MEF2C pathway (Figure 6E). WWP1 protein levels were significantly upregulated in the hearts of mice after 6 weeks of tail suspension and rhesus monkeys after 6 weeks of bed rest, and the protein levels of WWP1 and DVL2 were closely related to the development of cardiac atrophy through the CaMKII/HDAC4-dependent pathway. WWP1 knockout alleviated simulated microgravity induced-cardiac remodeling and function decline. The histological analysis demonstrated that WWP1 KO inhibited the decrease in the size of individual cardiomyocytes of mice after tail suspension. Moreover, the pathological cardiac remodeling signals, such as DVL2, CaMKII, and HDAC4, were activated in the heart of WT mice after tail suspension; however, WWP1 KO mice showed a different trend. Therefore, WWP1 represents a potential therapeutic target for cardiac remodeling and function decline induced by simulated microgravity.

Although myocardial hypertrophy and myocardial atrophy have opposite phenotypes, they show similar gene expression characteristics, such as “fetal” gene expression activation, supporting the concept that opposite changes in workload *in vivo* induce a similar transcriptional response (Depre et al., 1998). Compared with other forms of cardiac remodeling, little is known about the specific mechanism governing the microgravity-induced cardiac atrophy (Ling et al., 2018). To systematically explore the effects of spaceflight on the heart, Walls et al. (2020) used the Drosophila cardiac model to examine how prolonged exposure to microgravity affects cardiac health. They reported the effects of microgravity on heart function in WT fruit flies that were born, developed, and spent 1–3 weeks as adults aboard the ISS compared with their ground-based controls, the results showed that structural and functional cardiac remodeling occurs in response to microgravity; RNA sequencing and immunohistochemistry data suggested that alterations in proteostasis likely contribute to the observed changes in cardiac remodeling induced by microgravity (Walls et al., 2020). According to their research, cardiac atrophy mediated by protein homeostasis regulation may be a fundamental response of the heart muscle to microgravity (Walls et al., 2020). Protein ubiquitination is a multi-functional post-translational modification that affects a variety of disease processes, including cardiac remodeling (Willis et al., 2010). Our previous research found that E3 ubiquitin ligase WWP1 can stabilize DVL2 by catalyzing K27-linked polyubiquitination, and DVL2 positively correlated with WWP1 hypertrophic heart and mediated the regulation of the CaMKII/HDAC4/MEF2C axis in cardiac hypertrophy by WWP1 (Zhao et al., 2021). In this study, we found that the protein levels of WWP1 and DVL2 were significantly upregulated in the hearts of mice and rhesus monkeys after simulated microgravity, and WWP1 deficiency can alleviate simulated microgravity induced-cardiac remodeling by inhibition of the DVL2/CaMKII/HDAC4 pathway.

Well-characterized signaling molecules that regulate cardiac remodeling induced by pressure overload include DVL2 (Zhao et al., 2021), CaMKII (Feng and Anderson, 2017), and HDAC4 (Ling et al., 2012). DVL2 is highly evolutionarily conserved and participates in canonical and non-canonical Wnt pathways (Sharma et al., 2018). In particular, DVL2 acts as an activator in pressure overload-induced cardiac hypertrophy. CaMKII is an important regulator of non-canonical Wnt signaling, whose continuously activated form is critical in pathological cardiac remodeling (Gentzel et al., 2015; Feng and Anderson, 2017). The activation of CaMKII directly phosphorylates HDAC4, causing its relocalization to the cytoplasm and activation of MEF2C (Passier et al., 2000; Ling et al., 2012; Kreusser and Backs, 2014). The protein level of DVL2 and the phosphorylation level of CaMKII were increased in the heart of WT mice, but not WWP1 KO mice, subjected to tail suspension. The phosphorylation level of HDAC4 was similar to that of CaMKII (Figure 6A). In summary, we have discovered that WWP1 is involved in the regulation of simulated microgravity-induced cardiac remodeling through the DVL2/CaMKII/HDAC4/MEF2C pathway; moreover, WWP1 has potential as a therapeutic target for cardiac remodeling induced by simulated microgravity.

## Data Availability Statement

The original contributions presented in the study are included in the article/supplementary material, further inquiries can be directed to the corresponding author/s.

## Ethics Statement

The animal study was reviewed and approved by Animal Care and Use Committee of China Astronaut Research and Training Center.

## Author Contributions

YL and SL conceptualized the study. GZ, ZL, and XY made significant contributions in the methodology. GZ performed the validation and wrote the original draft. GZ and SL conducted the formal analysis. GZ, DZ, JL, ZL, JP, YZ, and WX performed the investigation. GZ, YL, and SL reviewed and edited the manuscript and acquired funding for the study. All authors contributed to the article and approved the submitted version.

## Conflict of Interest

The authors declare that the research was conducted in the absence of any commercial or financial relationships that could be construed as a potential conflict of interest.

## Publisher’s Note

All claims expressed in this article are solely those of the authors and do not necessarily represent those of their affiliated organizations, or those of the publisher, the editors and the reviewers. Any product that may be evaluated in this article, or claim that may be made by its manufacturer, is not guaranteed or endorsed by the publisher.
